# Association between psychotropic drug prescription and suicide rates in Scotland: population study

**DOI:** 10.1192/bjb.2021.88

**Published:** 2023-04

**Authors:** Fhionna R. Moore, Mairi Macleod, Trevor A. Harley

**Affiliations:** 1 Institute of Health and Wellbeing, School of Medical, Veterinary, and Life Sciences, University of Glasgow, UK; 2 School of Psychology, University of Dundee, UK

**Keywords:** Suicide, antidepressants, antipsychotics, anti-anxiety drugs, epidemiology

## Abstract

**Aims and method:**

Rates of prescriptions of antidepressants and suicide are inversely correlated at an epidemiological level. Less attention has been paid to relationships between other drugs used in mental health and suicide rates. Here we tested relationships between prescriptions of anxiolytics and antipsychotics and suicide rates in Scotland.

**Results:**

Suicide rates were inversely correlated with prescriptions of antidepressants and antipsychotics over 14 years (2004–2018), and positively with prescriptions of anxiolytics.

**Clinical implications:**

This illustrates the role of medications used in mental health in suicide prevention, and highlights the importance of identifying causal mechanisms that link anxiolytics with suicide.

Increased prescriptions of antidepressants worldwide have, at least at the population level, correlated with a reduction in suicide rates.^1^ For example, negative relationships have been reported between rates of prescription of antidepressants and population suicide rates in adults in the USA,^2^ Europe^3^ and Australia.^4^ This trend may, however, be moderated by gender, as female suicide rates were shown to be reduced with a corresponding increase in antidepressant sales in Italy, whereas male rates increased.^5^

Other classes of drug used in mental health, such as those prescribed for psychotic illnesses (antipsychotics) or anxiety (anxiolytics, hypnotics, benzodiazepines and barbiturates), have received relatively less attention in relation to suicide rates. Although there is evidence that elevated prescription rates of antipsychotics for people with schizophrenia are associated with a reduction in suicide rates in this patient subgroup,^6^ effects on suicide rates more widely remain underexplored. As antipsychotics are prescribed for conditions other than psychosis (e.g. severe unipolar or bipolar depression), it is possible that antipsychotic prescription rates act on population suicide rates in a similar way to antidepressants. Furthermore, given a positive association between anxiety disorders and suicidality,^7^ prescription of medications used to treat anxiety may also reduce suicide rates.

Here we modelled relationships between prescriptions of antipsychotics, anxiolytics and antidepressants and monthly male and female suicide rates in Scotland from 2004 to 2018.

## Method

We assert that all procedures contributing to this work complied with the ethical standards of the relevant national and institutional committees on human experimentation and with the Helsinki Declaration of 1975, as revised in 2008. The research was approved by the University of Dundee Research Ethics Committee.

### Prescriptions of medications used in mental health

Dispensed quantities of medications from April 2004 to December 2018 were provided by NHS Scotland's Information Services Division (NHS-ISD), for British National Formulary (BNF) categories 4.1 Medications used in anxiety (hypnotics, anxiolytics and benzodiazepines, HAB), 4.2 Medications used in psychosis (antipsychotics) and 4.3 Medications used in depression (antidepressants). Dispensed quantities were operationalised as numbers of paid items (i.e. number of items for which cost was reimbursed), as these best reflect the activity and costs associated with prescribing and supply of medicines to patients in NHS Scotland over time and is therefore the standard unit of measurement of prescriptions used by NHS-ISD. These data were freely available from NHS-ISD.

### Suicide rates

Numbers of all probable male and female deaths by suicide each month in Scotland from April 2004 to December 2018 were obtained from the Scottish National Records Office. Data represent date of death, rather than date of registration of death. These were made available once a confidentiality statement was signed. Cause of death was coded according to ICD cause-of-death guidelines^8^ (i.e. deaths coded as ‘intentional self-harm’ or ‘events of undetermined intent’). In 2011, the way in which deaths from ‘acute intoxication’ were classified changed. That is, some deaths that would previously have been counted under ‘mental and behavioural disorders due to psychoactive substance use’ were now counted under ‘poisoning’, which increased the number of deaths coded under ‘events of undetermined intent’. Therefore, all analyses were conducted using pre-2011 coding rules to ensure consistency in the ways in which deaths were coded as suicide for the years included in the study. Male and female monthly suicide rates per 100 000 were computed based on the median annual population estimate for 2004–2018 (*n* = 5.2 million; www.nomisweb.co.uk).

### Analyses

Moving averages over 3-month blocks were computed for prescription and suicide rates in order to reduce the impact of anomalous variation in rates across relatively short time periods. We then fit bivariate linear regression models separately for men and women to test the hypotheses that rates of prescription of HAB, antipsychotics and antidepressants predict male and female suicide rates. All analyses were conducted with IBM SPSS Version 22 for Windows.

## Results

For descriptive statistics see Table 1.

**Table 1:** Bivariate linear regression statistics for relationships between male and female suicide rates and numbers of paid items of medications used in anxiety, psychosis and depression over 3-month moving averages

	HAB prescriptions	Antipsychotic prescriptions	Antidepressant prescriptions
Prescription rate, mean (s.d.)	172 741.24 (4698.79)	67 314.2 (10 920.32)	40 9770.32, (45 782.31)
Female suicide rate (per 100 000): mean = 0.32, s.d. = 0.05	Adj. *R*^2^ = 0.05	Adj. *R*^2^ = 0.07	Adj. *R*^2^ = 0.07
	*F*^1,174^ = 10.65	*F*^1,174^ = 13.08	*F*^1,174^ = 14.65
	β = 0.24*	β = −0.27**	β = −0.28**
Male suicide rate (per 100 000): mean = 0.88, s.d. = 0.11	Adj. *R*^2^ = 0.16	Adj. *R*^2^ = 0.19	Adj. *R*^2^ = 0.24
	*F*^1,174^ = 35.25	*F*^1,174^ = 41.65	*F*^1,174^ = 54.74
	β = 0.41**	β = −0.44**	β = −0.49**

HAB, hypnotics, anxiolytics and benzodiazepines, prescribed for anxiety; adj., adjusted.

*P < 0.005, **P < 0.001.

Prescriptions of all three categories of medication significantly predicted male and female suicide rates (Table 1). Prescriptions of antipsychotics were related to reduced suicide rates in men (β = −0.44, *P* < 0.001) and women (β = −0.27, *P* < 0.001), as were prescriptions of antidepressants (men: β = −0.49, *P* < 0.001; women: β = −0.28, *P* < 0.001). Prescriptions of HAB, however, were associated with increased suicide rates in men (β = 0.41, *P* < 0.001) and women (β = 0.24, *P* < 0.005).

## Discussion

Rates of prescription of antidepressants and antipsychotics predicted lower male and female suicide rates over 14 years in Scotland. Rates of prescription of HAB, however, predicted higher male and female suicide rates.

Our results are consistent with previous research which has shown that population-level prescription rates of antidepressants are associated with reductions in suicide rates.^1–4^ Unlike Barbui et al,^5^ however, we found this to be the case for both men and women. The role of antidepressants in suicide prevention must be considered alongside evidence that suggests a potential risk of increased suicidal behaviour following commencement on antidepressants, particularly among children and adolescents.^9^ Our data were for all probable suicides and were not classified by age, so it is not possible to determine whether separate patterns exist across adults and children and adolescents.

We found prescription rates of antipsychotics to be related to reduced suicide rates for both men and women. This is, to our knowledge, among the first evidence to suggest that prescription of antipsychotics reduces population-level suicide rates, extending the relationship beyond suicide rates among people with schizophrenia.^6^ Given the use of antidepressants and antipsychotics in depression, it is perhaps not surprising that both are shown to reduce suicide rates in our sample.

Prescriptions of HAB were found to be positively related to suicide rates. That is, months with higher rates of prescription of HAB also had higher rates of male and female deaths by suicide. This is perhaps surprising given the association between anxiety disorders and suicidality.^7^ One possibility is that HAB are prescribed for a variety of difficulties, including the spectrum of anxiety disorders and insomnia, which may be chronic (e.g. as in generalised anxiety disorder or obsessive–compulsive disorder) or acute (e.g. insomnia linked to grief or a recent traumatic experience). This means that prescriptions of HAB may be more closely linked to incidence of acute distress (e.g. grief) and associated anxiety in the population than prescriptions of antipsychotics or antidepressants.^7,10^ Alternatively, hypnotics have been shown to correlate with increased risk for suicide by increasing suicidal ideation.^10^

Our results are consistent with those of Lodhi & Shah,^11^ who demonstrated that the association between suicide and prescription rates in an elderly population was negative for prescriptions of selective serotonin reuptake inhibitor antidepressants, antimanics, antipsychotics and analgesics, and positive for monoamine oxidase inhibitor antidepressants and hypnotics, anxiolytics and barbiturates. Taken with our findings in the general Scottish population, then, there is growing evidence that HAB are associated with increased suicide rates.

Our study was conducted across a relatively small geographical area and across a relatively brief period of time. Therefore we cannot be certain that results will translate to larger areas or longer periods. Our study cannot tell us anything about the likelihood of an individual man or woman dying by suicide as a result of their use of medications. We also cannot conclude, or infer without speculation, causal mechanisms from our correlational results and it is possible that unmeasured variables account for the relationships we have demonstrated.

Given our finding that rates of male and female deaths by suicide are positively related to prescriptions of HAB, we argue that future work should seek to identify the extent to which this relationship replicates across populations, as well as mechanisms by which HAB may contribute to suicidality.

## Data Availability

The data that support the findings of this study are available from the corresponding author (F.R.M.) on reasonable request.

## About the authors

**Fhionna R. Moore** was a senior lecturer in the School of Social Science, University of Dundee, at the time this work was carried out. She is now a trainee clinical psychologist in the College of Medical, Veterinary & Life Sciences, University of Glasgow, UK and with NHS Highland, Raigmore Hospital, Inverness, UK. **Mairi Macleod** is a research assistant in the School of Social Science, University of Dundee, UK. **Trevor. A. Harley** is a professor in the School of Social Science, University of Dundee, Dundee, UK.

## Supplementary material

For supplementary material accompanying this paper visit https://doi.org/10.1192/bjb.2021.115. click here to view supplementary material

## References

[1] Ludwig J, Marcotte DE, Norberg K. Anti-depressants and suicide. J Health Econ 2009; 28: 659–76. 19324439 10.1016/j.jhealeco.2009.02.002

[2] Gibbons RD, Hur K, Bhaumik DK, Mann JJ. The relationship between antidepressant prescription rates and rate of early adolescent suicide. Am J Psychiatry 2006; 163: 1898–904. 17074941 10.1176/ajp.2006.163.11.1898

[3] Gusmão R, Quintão S, McDaid D, Arensman E, Van Audenhove C, Coffey C, Antidepressant utilization and suicide in Europe: an ecological multi-national study. PLoS One 2013; 8(6): e66455. 23840475 10.1371/journal.pone.0066455 PMC3686718

[4] Hall WD, Mant A, Mitchell PB, Rendle VA, Hickie IB, McManus P. Association between antidepressant prescribing and suicide in Australia, 1991–2000: trend analysis. BMJ 2003; 326: 1008. 12742921 10.1136/bmj.326.7397.1008 PMC154757

[5] Barbui C, Campomori A, D'Avanzo B, Negri E, Garattini S. Antidepressant drug use in Italy since the introduction of SSRIs: national trends, regional differences and impact on suicide rates. Soc Psychiatry Psychiatr Epidemiol 1999; 34: 152–6. 10327841 10.1007/s001270050127

[6] Haukka J, Tiihonen J, Härkänen T, Lönnqvist J. Association between medication and risk of suicide, attempted suicide and death in nationwide cohort of suicidal patients with schizophrenia. Pharmacoepidemiol Drug Saf 2008; 17: 686–96. 18327869 10.1002/pds.1579

[7] Kanwar A, Malik S, Prokop LJ, Sim LA, Feldstein D, Wang Z, The association between anxiety disorders and suicidal behaviors: a systematic review and meta-analysis. Depress Anxiety 2013; 30: 917–29. 23408488 10.1002/da.22074

[8] World Health Organization. International Classification of Diseases,Tenth Revision (ICD-10). WHO, 2010.

[9] Cipriani A, Furukawa TA, Salanti G, Geddes JR, Higgins JP, Churchill R, Comparative efficacy and acceptability of 12 new-generation antidepressants: a multiple-treatments meta-analysis. Lancet 2009; 373: 746–58. 19185342 10.1016/S0140-6736(09)60046-5

[10] McCall WV, Benca RM, Rosenquist PB, Riley MA, McCloud L, Newman JC, Hypnotic medications and suicide: risk, mechanisms, mitigation, and the FDA. Am J Psychiatry 2017; 174: 18–25. 27609243 10.1176/appi.ajp.2016.16030336 PMC5205566

[11] Lodhi LM, Shah A. Psychotropic prescriptions and elderly suicide rates. Med Sci Law 2004; 44: 236–44. 15296248 10.1258/rsmmsl.44.3.236

